# Investigation on the Stability of Random Vortices in an Ion Concentration Polarization Layer with Imposed Normal Fluid Flow

**DOI:** 10.3390/mi11050529

**Published:** 2020-05-22

**Authors:** Jihye Choi, Ali Mani, Hyomin Lee, Sung Jae Kim

**Affiliations:** 1 Department of Electrical and Computer Engineering, Seoul National University, Seoul 08826, Korea; onnees@gmail.com; 2 Department of Mechanical Engineering, Stanford University, Stanford, CA 94305, USA; alimani@stanford.edu; 3 Department of Chemical and Biological Engineering, Jeju National University, Jeju 63243, Korea; 4 Inter-University Semiconductor Research Center, Seoul National University, Seoul 08826, Korea; 5 Nano Systems Institute, Seoul National University, Seoul 08826, Korea

**Keywords:** ion concentration polarization, vortex, instability, normal flow

## Abstract

While nanoscale electrokinetic studies based on ion concentration polarization has been actively researched recently, random vortices naturally occur, leading to significantly destabilize in laboratory experiments or practical applications. These random vortices agitate the fluid inside microchannels and let the sample molecules seriously leak out preventing them from being controlled. Therefore, several trials have been reported to regulate those uninvited fluctuations by fluid flow tangential to a nanoporous membrane. Indeed, the influence of normal flow should be studied since the mass transport happens in the normal direction to the membrane. Thus, in this work, the nonlinear influence of normal flow to the instability near ion-selective surface was investigated by fully-coupled direct numerical simulation using COMSOL Multiphysics. The investigation on the effect of normal flow revealed that a space charge layer plays a significant role in the onset and growth of instability. The normal flow from the reservoir into the ion-selective surface pushed the space charge layer and decreased the size of vortices. However, there existed a maximum point for the growth of instability. The squeeze of the space charge layer increased the gradient of ion concentration in the layer, which resulted in escalating the velocity of vortices. On the other hand, the normal flow from the ion-selective surface into the reservoir suppressed the instability by spreading ions in the expanding space charge layer, leading to the reduction of ion concentration delayed the onset of instability. These two different mechanisms rendered asymmetric transition of stability as a function of the *Peclet* number and applied voltage. Therefore, this investigation would help understand the growth of instability and control the inevitable random vortices for the inhibition of fluid-agitation and leakage.

## 1. Introduction

Electro-convective instability near an ion-selective surface on concentration polarization has received great attention since it plays critical roles in electrodialysis [1], separation [2,3,4] and preconcentration of charged molecules [5,6,7] at over-limiting regime. Perturbation of salt concentration along an ion-selective surface triggers Coulombic force-driven fluid motion, leading to the generation of random vortices [8]. These vortices bring ions from ion-abundant bulk to an ion-depleted region in the vicinity of the surface and are grown chaotically [9,10]. The appearance of non-linear response beyond a diffusion-limited transport, known as over-limiting current, is accompanied by this instability [11]. Thus, over-limiting current has improved mass transport in several electrochemistry applications including ion-selective surfaces. In the meantime, the depletion of ions observed in over-limiting regime has enabled desalination [12,13,14] and preconcentration of charged particles [15,16,17]. Yet, highly strong random-vortices can let the depleted layer fluctuate and even destabilize the entire system near an ion-selective surface. Kim et al. witnessed that irregular vortices were excessively fluctuating with tangential flow admitted from the reservoir to depletion region, causing the leakage of preconcentrated charged particles [18]. This study inferred that the distribution of ions inside depletion zone was also irregular. In general, it was a dilemma for experimentalists of concentration polarization; (i) increasing voltage or current beyond limiting-regime inevitably initiated random vortices, which agitated the fluid inside microchannels, disturbing preconcentration of particles or purity of desalination, (ii) applying an imposed flow over the ion-selective membrane either in normal or tangential to the membrane to suppress the random vortices or to increase the amount of processed fluid from the microchannel without unwanted leakages. Accordingly, it has been tried to control the vortices through geometrical restriction of microchannels [2,19,20].

There have been a couple of studies investigated the dynamics of the vortices when the imposed flow affects them. Ehlert et al. observed the thinning of depletion zone near a circular ion-selective membrane depending on different shear flow rates when the pressure-driven flow was applied [21]. In Kwak et al.’s study, the behavior of electro-convection under shear fluid flow was investigated both in experiment and numerical simulation [22]. Those studies discussed the effect of the imposed flow which was only tangential to the ion-selective surface. In practical applications, extraction step of processed fluid usually exploits the injection of external flow tangential to ion-selective surfaces. Conventionally, the water permeability of ion exchange membrane was treated as negligible parameter in the analysis of electrodialysis and micro/nanofluidic devices utilizing concentration polarization phenomenon. However, several studies have revealed that the imposed normal flow significantly altered the ion transportation through the membrane, leading to distinguishable concentration polarization behavior [23,24,25]. In addition to these studies, one configuration of nanoelectrokinetic biomolecular preconcentrators utilized the external flow normal to the nanoporous membrane for easiness of operation [20,26,27,28,29]. For studying the characteristics of such situations, how the normal flow affect electroconvective instability was investigated in this work. Although convectional membranes have *O*(10^−2^)–*O*(10) of Peclet number (this value represents characteristic magnitude of normal flow), we set stronger normal flow for theoretical investigation of nonlinear interactions between the normal flow and electroconvection.

In parallel, it is also important to understand the effect of normally imposed flow on electro-convection. This is because mass is indeed transported through an ion-selective surface in normal direction to the surface. In the analysis of transport phenomena at the interface of electrolyte/nanoporous membrane, it was reported that water-permeance through ion-selective membrane affected ion and particle transportation near ion-selective membrane [24]. Bruggen et al. reviewed that nanofiltration membrane, which selectively sieves charged ions, has permeability of *O*(10^−2^) to *O*(1) in the Peclet number (*Pe*) in general [30]. Recently, ultrafast molecule sieving nanosheet membranes was fabricated with *Pe*~*O*(10) [31]. Therefore, water permeability through an ion-selective membrane is a non-negligible factor for the consideration of membrane process.

Attempts to analyze the influence of normal flow on concentration polarization were found both in experiments and theories. Li et al. experimentally measured the thickness of depletion layer under different normal electro-osmotic flow into sub-microchannels including nanochannels [11]. Tanaka et al. evaluated the relationship between concentration polarization and limiting current on desalting membranes when a normal flow imposed to the membrane, concluding that natural convection necessarily becomes driving force for the redistribution of ion concentration in a depletion layer [32]. Theoretical work was performed by Hsu et al. that the convective flow of fluid perpendicular to ion-selective surface improved current efficiency at under-limiting regime [33]. However, most practical applications conventionally occur at over-limiting regime to enhance mass transport by increasing drift and convective migrations. Here, electro-convection at over-limiting current is a main key point to understand the instability inside a depleted layer. Khair paid attention to this importance and investigated the role of admitted normal flows on second-kind electro-osmotic instability when concentration polarization occurs [34]. Linear instability of the concentration polarization was asymptotically examined when a normal flow is imposed to an ion-selective surface. In his work, *Pe* of normal flow was *O*(1), voltage was *O*(10) and the domain was electroneutral. He found a marginal stability curve using second-kind electro-osmotic slip at an ion-selective surface proposed by Rubinstein and Zaltzman [8]. Although Khair’s work revealed the important interaction between the electro-convective instability and the imposed normal flow through asymptotic analysis, he neglected the finite thickness of electrical double layer (EDL) and space charge layer (SCL) nearby the ion-selective surface. Recent simulation studies [35,36] have suggested that the dynamical changes of EDL and SCL can significantly affect the dynamics of electro-convective instability; onset voltage, instability growth rate and current-voltage relations, etc. Thus, instead of the asymptotic approach, the fully-coupled analysis for the instability should be needed.

In this work, the non-linear influence of normal flow to the instability near ion-selective surface was investigated by fully-coupled direct numerical simulation using COMSOL Multiphysics for the chaotic transport system with a finite EDL and SCL. An ion-selective surface in a domain admitted each normal flow into an ion-selective membrane and out of the membrane, and the velocity of the electro-convection was analyzed to explore the effect of imposed normal. A marginal stability curve was numerically obtained which had asymmetric transition from stable to unstable state depending on the direction of imposed normal flow. 

## 2. Numerical Formulation

### 2.1. Domain Description

**Figure 1:**
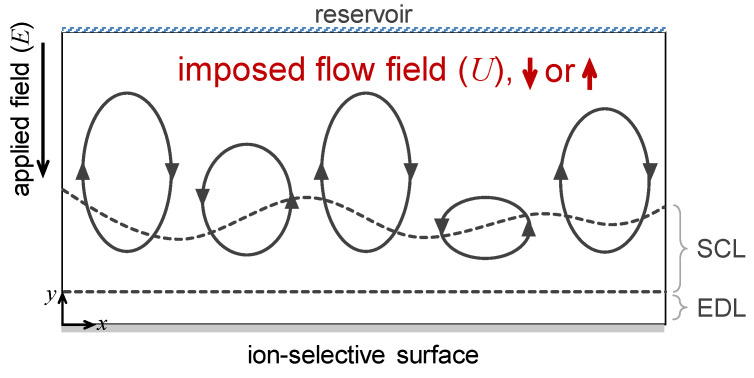
Schematic of numerical domain. The characteristic length scale was 1 for the *y*-directional length of the domain, while it was 4 for the *x*-directional one. The reservoir was assumed to have uniform ion concentration. The ion-selective surface was water-permeable, through which only cations and fluid flow can pass. The periodic boundary condition described an infinite domain case in horizontal direction.

Numerical domain was schematically represented in Figure 1. The *y*-directional length of the domain was chosen as characteristic length scale, 1, and the *x*-directional length of the domain was as set to be 4. From the literature [36], the dimensionless spanwise-width should exceed 2 to avoid spurious numerical results for periodic domain. The numerical domain had three types of boundary: reservoir, ion-selective surface and periodic boundaries. The reservoir was assumed to have uniform ion concentration. The ion-selective surface was water-permeable through which only cations and fluid flow can pass. The periodic boundary condition described an infinite domain case in horizontal direction. 

When the electric field was applied in the direction from the reservoir to ion-selective surface, concentration polarization phenomenon occurred, leading to the formation of SCL and mixing layer (ML) above EDL. The fluctuation of small-amplitude inside SCL would result chaotic fluid motions which are electro-convective instability. While the electro-convection existed, we simultaneously considered uniform imposed normal flow at the reservoir and the ion-selective surface.

### 2.2. Governing Equations and Boundary Conditions

The fully-coupled chaotic motion was described by the Poisson equation for electromagnetism, the Nernst-Planck equation for mass transfer, the continuity equation for mass conservation of fluid and the Stokes equations with the Coulombic body force for momentum transfer as follows:

(1) $$-\nabla^{2}\phi =\frac{1}{2{\lambda_{D}}^{2}}\left(c^{+}-c^{-}\right),$$

(2) $$\frac{\partial c^{\pm }}{\partial t}=-\nabla \cdot \left(c^{\pm }u-\nabla c^{\pm }\mp c^{\pm }\nabla \phi \right),$$

(3) ∇⋅u=0 and

(4) $$\frac{1}{Sc}\frac{\partial u}{\partial t}=-\nabla p+\nabla^{2}u-\frac{\kappa }{2{\lambda_{D}}^{2}}\left(c^{+}-c^{-}\right)\nabla \phi .$$

Each equation was non-dimensionalized, where *ϕ* is electric potential, *λ_D_* is the dimensionless Debye length, *c*^+^ was cation concentration, *c*^−^ was anion concentration, *t* was the time, **u** was velocity of fluid, *Sc* was the Schmidt number, *p* was pressure, and *κ* was the electrohydrodynamic coupling constant defined as

(5) $$\kappa =\frac{\tilde{\varepsilon }{{\tilde{V}}_{T}}^{2}}{\tilde{\mu }\tilde{D}}$$

The tilde (‘~’) meant dimensional quantity. The characteristic scales were denoted in Table 1. $\tilde{\varepsilon }$ was the electrical permittivity of fluid, ${\tilde{V}}_{T}$ was the thermal voltage scale, $\tilde{\mu }$ was the fluid viscosity and $\tilde{D}$ was the diffusivity of electrolyte. For simplicity, the cation and anion diffusivities were assumed to be the same (i.e., KCl electrolyte). The actual values of KCl electrolyte are 1.96 × 10^−9^ m^2^/s for K^+^ and 2.03 × 10^−9^ m^2^/s for Cl^−^ [37] so that the diffusivities of two ionic species were treated as 2.00 × 10^−9^ m^2^/s. The *κ* was typically *O*(1) so that we set *κ* = 0.5, which was the same value in Druzgalski’s work [36]. In order to consider the effect of finite thickness of EDL and SCL, *λ_D_* was fixed to be 0.001. This case was corresponded with *O*(100 μm) gap filling with *O*(10 μM) of electrolyte.

The coupled-governing equations were simultaneously solved with following boundary conditions by the finite element method in COMSOL Multiphysics. At reservoir interface, the dimensionless electric potential was set to be 25, 50, 75, 100 and 125. Cation and anion concentration were equally given as 1. Both on the ion-selective surface and reservoir side, the *y*-component of fluid velocity (i.e., imposed normal flow) was given as fixed values from −50 to 50 (i.e., ±50, ±40, ±30, ±20, ±10 and 0). These normal flow velocities were represented as the Peclet number ($Pe={\tilde{U}}_{n}{\tilde{L}}_{y}/\tilde{D}$), where ${\tilde{U}}_{n}$ is the dimensional quantity of the normal flow speed). The negative sign of velocity meant that the normal flow was admitted in the direction from the reservoir into the ion-selective surface (downward), while the positive sign of velocity meant that the normal flow was admitted in the direction from the ion-selective surface into the reservoir (upward). At the ion-selective surface, we employed the ideal cation-selectivity conditions; the Donnan potential (the Donnan concentration for cation and zero anionic flux), Additionally, the imposed normal flow was applied. The time-dependent solution of *ϕ*|_reservoir_ = 25 was obtained from *t* = 0 to *t* = 2 and other cases of reservoir potential were calculated from *t* = 0 to *t* = 0.2 [38]. The above governing equations and boundary conditions were solved by the commercial software, COMSOL 4.4.

The Peclet number or flow rate through the membrane can be directly expressed by measurable membrane properties. According to the Darcy’s law, the flow rate ($\tilde{Q}$) can be characterized by the water permeability (${\tilde{k}}_{w}$) of the membrane. Its dimensional form is

(6) $$\tilde{Q}=-\frac{{\tilde{k}}_{w}}{\tilde{\mu }}\tilde{\nabla }\tilde{p}$$

In terms of the dimensionless form,

(7) $$Pe=-\frac{k_{w}}{A_{mem}}\nabla p\approx -\frac{k_{w}\Delta p}{A_{mem}d_{mem}}$$

where *A_mem_* is the cross-sectional area of the membrane, *d_mem_* is the membrane thickness, and water permeability is normalized by ${{\tilde{L}}_{y}}^{4}$. Note that the Δ*p* represents the pressure difference across the membrane. Thus, the Peclet number (or flow rate) used in this work can be directly related to membrane properties, such as *A_mem_*, *d_mem_*, and *k_w_*.

## 3. Results and Discussions

### 3.1. The Effect of Space Charge Layer on Electro-Convection with Admitted Normal Flow

One dimensional mass transport through an ion-selective surface with uniform fluid flow normal to the surface had been investigated at over-limiting regime of concentration polarization phenomenon [34]. In the work, one of main conclusion was asymptotic analysis using electro-osmotic slip condition revealed that normal fluid flow with *Pe*~*O*(1) and *ϕ*~*O*(10) helps grow or suppress instability. The fluid flow in the direction from reservoir into the ion-selective surface prevented the expansion of vortices arising from random perturbation near ion-selective surface, suppressing the growth of instability. In the case of fluid flow in opposite direction, the instability was enhanced by pulling the instability in the same direction of the fluid flow. 

**Figure 2:**
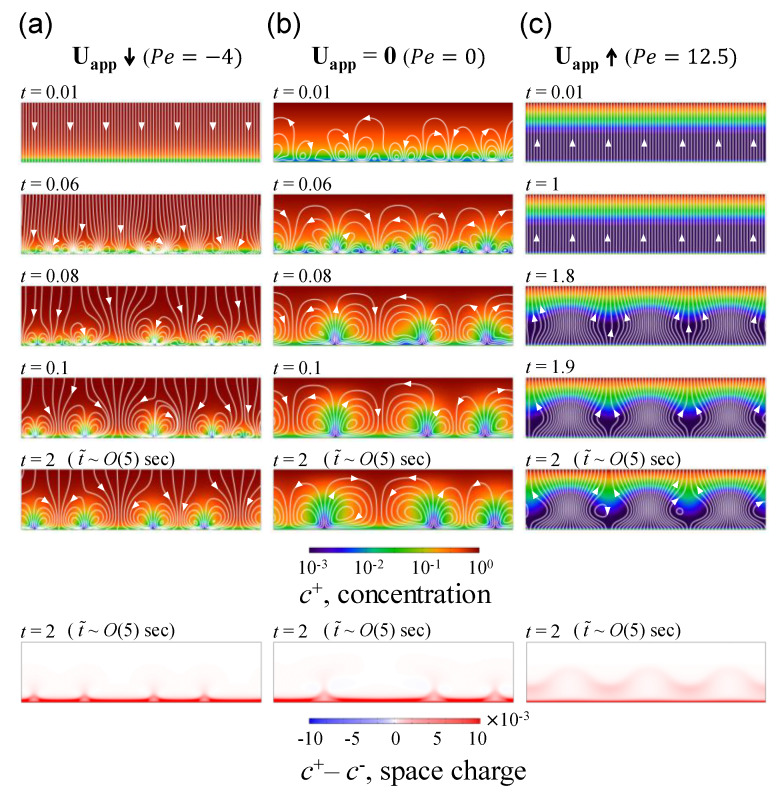
Cation concentration according to each time lapse and the ion concentration of space charge layer (SCL) when (**a**) the fluid flow was imposed from the reservoir into the ion-selective surface (*Pe* = −4), and (**b**) there was no fluid flow (*Pe* = 0) and (**c**) the fluid flow was imposed from the ion-selective surface into the reservoir (*Pe* = 12.5). The white lines represent stream line and the white arrows indicate the direction of the flow. Note that the ion concentrations were rendered in a log scale and denoted time was dimensionless. In order to avoid confusion between dimensional and dimensionless variables, we notated dimensional time ($\tilde{t}$) at the case of *t* = 2. This value was corresponded with the case of *O*(100) μm gap filling with *O*(100) μM KCl electrolyte.

Here, we conducted fully-coupled numerical simulation under rigorous consideration of finite EDL and SCL regarding the influence of normally imposed fluid flow on the instability near an ion-selective surface. As shown in Figure 2, the upper boundary of the domain represented a bulk reservoir in which low voltage (*ϕ* = 25) was applied, and the lower boundary of the domain represented a cation-selective surface. When normal flow was imposed in the direction from the reservoir to ion-selective surface as shown in Figure 2a (i.e., downward direction), the size of generated random vortices became smaller than the case without any imposed normal flow (Figure 2b). In align with the analysis of Khair’s work [34], the imposed flow into the ion-selective surface inhibited the propagation of random vortices toward the reservoir. Comparing the SCLs (i.e., *c*^+^–*c*^−^) in Figure 2a,b, the SCL was extremely close to the ion-selective surface so that it could be assumed to be near zero thickness, inferring much smaller thickness of EDL even we considered it as finite length. This explained the reason why we obtained the comparable consequences with Khair’s ones which employed second-kind electro-osmotic slip velocity [8]. When the direction of imposed flow was inversed (i.e., *Pe* = 12.5) but still in low voltage (*ϕ* = 25), the SCL appears as spaciously as the half of the domain as shown in Figure 2c. Accordingly, electro-convective vortices grew and expanded in the middle of the domain. Such deformation and thickness of SCL is non-negligible so that we received a hint that a finite SCL plays a crucial role in electro-convective instability. 

As shown in space charge plots in Figure 2, the upward normal flow can induce thicker SCL compared to other flow directions. However, the space charge density became to lessen with thicker SCL. It is noticeable that the regular intervals in the peak of SCL plots can be found the similar patterns in other simulation works [35,36] Although other simulation works conducted with zero-normal flow, the intervals were in *O*(1) spacing. This regularity is associated with vortex generation in the vicinity of membrane. Typically, the vortices were formed as a pair of small vortex so that its size can determine the regularity. Such regularity or vortex size was experimentally observed by Yossifon et al. [9] and was approximately proportional to the thickness of concentration polarization layer (~1.25*L_y_*). Note that the time steps in Figure 2a,b vs. Figure 2c were different due to a growth rate of instability which will be discussed in next section.

### 3.2. Growth Rate of Electro-Convective Instability

How the finite EDL and SCL affect instability was investigated through calculating the growth rate of the flow velocity. Imposed normal flow and electro-convective flow were mixed in the domain. In order to separate and discuss the growth rate of electro-convection, the flow field (**u**) was decomposed as

**u** = **U**_app_ + **u**_EC_, (8)

where **U_app_** is the imposed normal flow, and **u_EC_** is the flow velocity solely by the electro-convective instability. Note that the space- and temporal-average of $u_{\mathrm{EC}}$ in the entire domain was equal to be zero as similar as turbulence analysis [39]. Thus, we defined the domain-averaging root-mean-square velocity (*U_rms_*) of the electro-convective instability as

(9) $$U_{rms}=\frac{\int_{\Omega }\sqrt{u_{EC}\cdot u_{EC}}d\Omega }{\int_{\Omega }d\Omega }=\frac{\int_{\Omega }\sqrt{\left(u-U_{app}\right)\cdot \left(u-U_{app}\right)}d\Omega }{\int_{\Omega }d\Omega }$$

where Ω represented the domain. Positive **U_app_** described the normal flow away from the ion-selective surface (i.e., upward normal fluid flow), while negative **U_app_** does the normal flow into the ion-selective surface (i.e., downward normal fluid flow). Utilizing above decomposition and integration, the intensity of the chaotic fluid flow can be extracted apart from the imposed normal flow. The dynamics of electro-convective instability was investigated by the observation on the growth rate determined as the exponent of fitting curve of *U_rms_* as a function of time.

**Figure 3:**
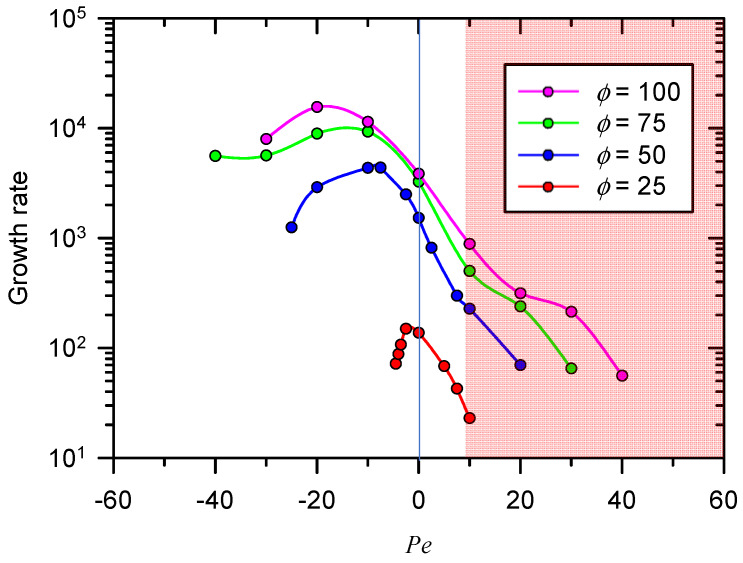
The plot of growth rate according to the *Pe*. The tendency of changes in growth rate was different depending on the direction of normal flow. When the *Pe* was positive (upward fluid flow), the growth rate decreased monotonically. There was a maximum point of the growth rate when the *Pe* was negative (downward fluid flow).

As shown in Figure 3, the tendency of changes in growth rate was different depending on the direction of normal flow. The positive growth rate means the electroconvective domain becomes unstable. In other words, the stable domain has negative growth rate. From the entire rage of *Pe* (i.e., −∞ < *Pe* < ∞), the domain transited through stable-unstable-stable so that the signs of growth rates were negative-positive-negative. In Figure 3, only several positive growth rates were plotted in log-scale to characterize local maximum so that its calculated range of *Pe* should be different according to the potential value. When the *Pe* was positive (upward flow flow), the growth rate decreased monotonically. The normal flow with positive *Pe* would spread the SCL into the bulk side, lowering the concentration gradient in the layer. This reduced gradient in the SCL was incapable of triggering the disturbance of fluid flow. Note that the growth rate would be saturated as the applied voltage increased. 

Interesting observation in the case of *Pe* < 0 was that the growth rate had the local maximum as a function of *Pe*. This inferred that the downward normal flow does not always suppress the growth of electro-convection, and indeed, a range of *Pe* which can enhance the growth rate existed. The range was extended as the applied voltage became higher. In the physical point of view, weak downward flow (i.e., small *Pe*) suppresses the expansion of SCL, which leads to the decrease of vortex size. Yet, the velocity of the vortices increases because the concentration gradient in the SCL becomes steeper (see Supplementary Information Note 1: SI Figure S1). This enhances the enhancement of instability growth rate when the weak downward flow is imposed.

However, despite the increment of the gradient, the growth rate eventually decreased as a function of *Pe*. This would be attributed to the dwindled size of vortices which replenished ions from the bulk side into the depletion layer. The strong downward fluid flow finally inhibited the expansion of electro-convective vortices toward reservoir, delaying the growth rate of instability. 

### 3.3. Rearrangement of Space Charge Layer and Mixing Layer Depending on Pe

**Figure 4:**
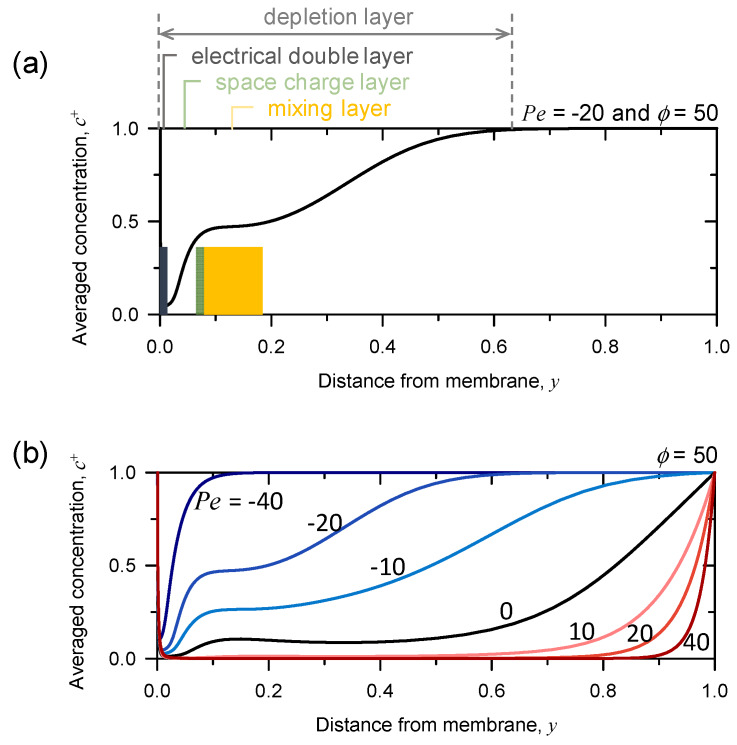
The different behavior of growth rate depending on the sign of *Pe* (*t* = 0.2) was attributed to the distribution of ion concentration in the domain. (**a**) Representative schematic for electrical double layer (EDL), SCL, and mixing layer (ML) (*Pe* = -20, *ϕ* = 50). (**b**) Averaged concentrations at *ϕ* = 50 with varying *Pe* from negative to positive.

The different behavior of growth rate depending on the sign of *Pe* (*t* = 0.2) was attributed to the distribution of ion concentration in the domain. Representatively, EDL, SCL, and ML were plotted in Figure 4a when *Pe* was −20 and the applied voltage was 50. The thickness of EDL was ~1.6 × 10^−2^ which was extremely close to the ion-selective surface. The SCL, where the averaged concentration of cation increased, was ~8.33 × 10^−2^ thick. The plateau of ion concentration beyond SCL was defined as a ML in which the vortices removed the gradient of ion concentration by strong mixing. The complete picture including all of the layers also confirmed the validity of our numerical scheme which considered the finite thickness of EDL and SCL, as previous literature described [36,40].

Figure 4b plotted averaged concentrations at *ϕ* = 50 with varying *Pe* from negative to positive. When *Pe* was equal to zero (i.e., no normal flow), the ML broadly expanded in the domain. Imposing upward normal flow (*Pe* > 0) reduced the overall averaged concentration in the domain, as well as the averaged concentration in the SCL, and it also lengthened ML, as we explained in the previous section. By revisiting the growth rate of instability when *Pe* was positive in Figure 3, it can be inferred with these results that the upward normal flow (*Pe* > 0) spread the ion concentration and let it sparse throughout the domain; thus, the perturbation of salt concentration was too weak to develop the effective fluid motion bringing electro-convection. In other words, the upward normal flow (*Pe* > 0) slowed down the onset of instability-growth, while it helped expanding the area where the vortices exist.

In the case of the downward normal flow, *Pe* < 0 (*ϕ* = 50), the slope of averaged concentration in the SCL became steep and the length of ML became shorter as the absolute value of negative *Pe* became larger. A simple physical interpretation was that the downward normal flow brought ions from the reservoir to replenish the depletion zone. The introduced ions would steepen the gradient of ion concentration in the SCL; thus, the growth of instability was enhanced by providing more chances to generate lateral non-uniform salt concentration. However, in the case of *Pe* = −40, the ML disappeared due to strong normal fluid flow. The strong fluid flow restrained the growth of vortex-seeds, failing to persist the development of instability with increasing the absolute value of *Pe* (*Pe* < 0), which was in-line with the observation of local maximum in the range of *Pe* < 0 (Figure 3).

### 3.4. Asymmetric Transition of Electro-Convective Instability

**Figure 5:**
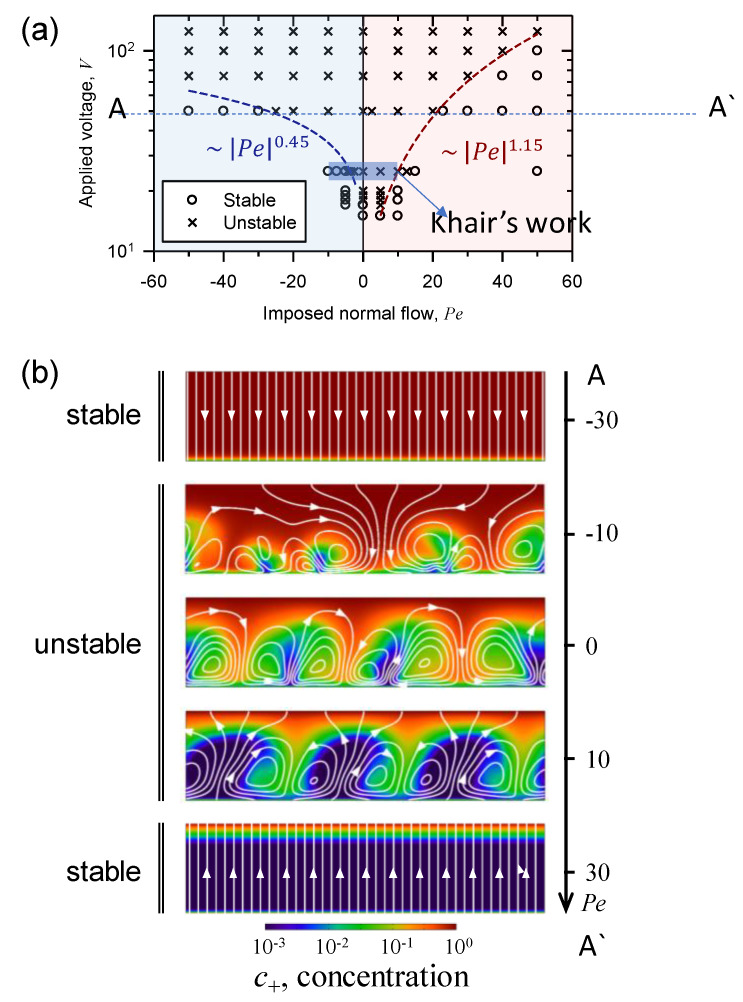
(**a**) A stability map was investigated as a function of both applied voltage and the properties of imposed normal fluid flow. A cross sign indicated that there existed vortices (unstable) and a circle sign indicated that there were no vortices in the domain (stable). Marginal stability curve (dotted line) was also obtained. In the case of positive *Pe*, the marginal curve followed ~ |*Pe*|^1.15^. In the case of negative *Pe*, the marginal curve followed ~ |*Pe*|^0.45^. (**b**) The distribution of ion concentration in the domain along increasing *Pe* from negative to positive value at *ϕ* = 50.

A stability map was investigated as a function of both applied voltage and the properties of imposed normal fluid flow as shown in Figure 5a. A cross sign indicated that there existed vortices (unstable) and a circle sign indicated that there were no vortices in the domain (stable). Marginal stability curve (dotted line) was also obtained. A threshold voltage for instability under no imposed normal flow was ~17.

Scaling analysis showed that the marginal curves were different depending on the sign of *Pe*. In the case of positive *Pe*, the marginal curve followed ~ |*Pe*|^1.15^. In the case of negative *Pe*, the marginal curve followed ~ |*Pe*|^0.45^. Larger absolute value of *Pe* was demanded to achieve a transition from unstable to stable state in the case of negative *Pe* than in the case of positive *Pe*. Figure 5b showed the distribution of ion concentration in the domain along increasing *Pe* from negative to positive value at *V* = 50. When normal flow was imposed in the direction from the reservoir into the ion-selective membrane (*Pe* < 0), the vortices shrank but instability enhanced until the downward normal flow removed mixing layer. The replenished ions provided by the downward normal flow increased overall concentration in the domain, yielding more chances to produce lateral perturbation near ion-selective surface in salt concentration. This helped the instability grow before the disappearance of mixing layer and vortices. This would contribute to the delay of the transition value of negative *Pe*. 

When the upward normal flow was imposed (*Pe* > 0), the size of vortices became large. However, the upward normal flow eventually washed out the ions in SCL toward the reservoir side. It resulted in the disappearance of SCL and the overall decrease of salt concentration in the domain. As the *Pe* further increased to ~*O*(10), the reduced ion concentration would be unable to create valid lateral perturbation, allowing faster stabilization of the instability. The numerical simulation revealed that the fluid flow in large *Pe* >> 1 lowered the ion concentration in SCL with finite thickness so that the growth of effective vortex-seeds was prevented. In brief, instability was existed but finally eliminated for both cases of normal flow in opposite directions as the absolute value of *Pe* increased; however, the asymmetric transition from unstable to stable state was originated from the different mechanism of imposed normal flow.

## 4. Conclusions

Theoretical studies have been challenging in nanoscale electrokinetic system, because one has to have full-understanding of electro-convective instability stems from the non-linear dynamics of electromagnetism/mass transfer/momentum transfer coupled system. In this work, the non-linear influence of normal flow to the instability near ion-selective surface was investigated by fully-coupled direct numerical simulation using COMSOL Multiphysics for the chaotic transport system with finite EDL and SCL. An ion-selective surface in a domain admitted each normal flow into an ion-selective membrane and out of the membrane, and the velocity of the electro-convection was analyzed to explore the effect of imposed normal. The analysis showed that SCL played a critical role in the onset of instability. The imposed fluid flow from the reservoir to the ion-selective surface (downward flow) squeezed the SCL preventing the growth of instability. One interesting observation was that the downward flow did not suppress the instability monotonically. There was a maximum point of *U_rms_* as the magnitude of downward flow increased. The analysis of energy spectrum by Fast Fourier transform revealed the reason that the velocity of vortices increased, while the size of them became small due to the decrease of the size of SCL. On the other hand, the imposed fluid flow from the ion-selective membrane to the reservoir (upward flow) spread out ions in SCL. Then, reduced charge density slowed down the appearance of vortices. As the magnitude of the upward flow became further larger, ions were washed out on a domain into the bulk side, resulting in the suppression of the instability. A marginal stability curve was numerically obtained. It was found that the transition from stable to unstable state was asymmetric depending on the direction of imposed normal flow. In the case of positive *Pe*, the marginal curve followed ~ |*Pe*|^1.15^. In the case of negative *Pe*, the marginal curve followed ~ |*Pe*|^0.45^. Those two distinct mechanisms yielded asymmetric transition of chaotic electro-convection near water-permeable ion-selective surface. 

According to previous literatures, three basic mechanisms for over-limiting regime in ion concentration polarization phenomenon are surface conduction, convection by electro-osmotic flow and electro-osmotic instability [41,42,43,44]. The marginal map obtained here would provide the range of experimental conditions for suppressing the instability among the three basic mechanisms. This would help the control of inevitable random vortices for the inhibition of fluid-agitation and unwanted leakage.

## Figures and Tables

**Table 1:** Characteristic scales used in this work. The tilde (‘~’) meant dimensional quantity. $\tilde{D}$ is the diffusivity of electrolyte, $\tilde{R}$ is the gas constant, $\tilde{T}$ is the absolute temperature, and $\tilde{\mu }$ is the fluid viscosity.

Physical Quantity	Characteristic Scale	Description
Length	${\tilde{L}}_{y}$	*y*-directional length of numerical domain
Time	${\tilde{\tau }}_{D}=\frac{{\tilde{L}y}^{2}}{\tilde{D}}$	Diffusion time scale
Electric potential	${\tilde{V}}_{T}=\frac{\tilde{R}\tilde{T}}{\tilde{F}}$	Thermal voltage
Concentration	${\tilde{c}}_{0}$	Bulk concentration
Pressure	$\frac{\tilde{\mu }\tilde{D}}{{\tilde{L}y}^{2}}$	Diffusion-scaled pressure
Flow velocity	${\tilde{U}}_{0}=\frac{\tilde{D}}{{\tilde{L}}_{y}}$	Diffusion-scaled velocity
Current density	${\tilde{i}}_{0}=\frac{\tilde{F}\tilde{D}{\tilde{c}}_{0}}{{\tilde{L}}_{y}}$	Diffusion-limited current density

## Supplementary Materials

The following are available online at https://www.mdpi.com/2072-666X/11/5/529/s1, Figure S1: Energy spectrum of random vortices at the edge of the EDL is plotted according to the wave number when (a) the *Pe* was negative (downward flow) and (b) the *Pe* was positive (upward flow).
