# Supplementary material for: Investigation on the Stability of Random Vortices in an Ion Concentration Polarization Layer with Imposed Normal Fluid Flow
Source: Micromachines (Basel). 2020 May 22;11(5):529. doi: 10.3390/mi11050529 (PMC7281587; doi:10.3390/mi11050529)
Supplement: Supplementary file 1 [file micromachines-11-00529-s001.pdf]

micromachines

Supplementary Materials: Investigation on the Stability of Random Vortices in an Ion Concentration Polarization Layer with Imposed Normal Fluid Flow

Jihye Choi [1], Ali Mani [2], Hyomin Lee [3,*] and Sung Jae Kim [1,4,5,*]

[1] Department of Electrical and Computer Engineering, Seoul National University, Seoul 08826, Republic of Korea; onnees@gmail.com (J.C.)
[2] Department of Mechanical Engineering, Stanford University, Stanford, CA 94305, USA; alimani@stanford.edu (A.M.)
[3] Department of Chemical and Biological Engineering, Jeju National University, Jeju 63243, Republic of Korea
[4] Inter-University Semiconductor Research Center, Seoul National University, Seoul 08826, South Korea
[5] Nano Systems Institute, Seoul National University, Seoul 08826, South Korea
* Correspondence: gates@snu.ac.kr (S.J.K.); fluid@jejunu.ac.kr (H.L.)

Received: 28 April 2020; Accepted: 20 May 2020; Published: date

Supplementary Note 1. The energy spectrum according to the direction of imposed normal flow

The energy of convection can be analyzed by Fourier transform as shown in Supplementary Figure 1. Those plots are the energy spectrum calculated by Fast Fourier transform of the x-component of velocity on $y = 0.001$ (at the edge of Debye length). The energy spectrum, E, was the averaged energy spectrum from $t = 0.1$ to $t = 0.2$.

When the applied voltage was 50 and the Pe was negative (Supplementary Figure S1a), the energy became large and then went to zero as the magnitude of the Pe became large. It is aligned with the result that there was a maximum point for U_{rms} with negative Pe in Figure 3. Comparing $Pe = 0$ and $Pe = -20$, energy increased although the fluid flow prevented the expansion of a space charge layer, which means the instability grew. Especially, the convection with large wave number contributed the increment of energy. This represented that the size of vortices became small but the velocity increased. This explained why the instability grew in spite of the decrease of the size of space charge layer. On the other hand, the energy decreased monotonically as the fluid flow was imposed from the ion-selective membrane to the reservoir (upward flow) as shown in Supplementary Figure S1b. This was the same result with the results in Figure 3.

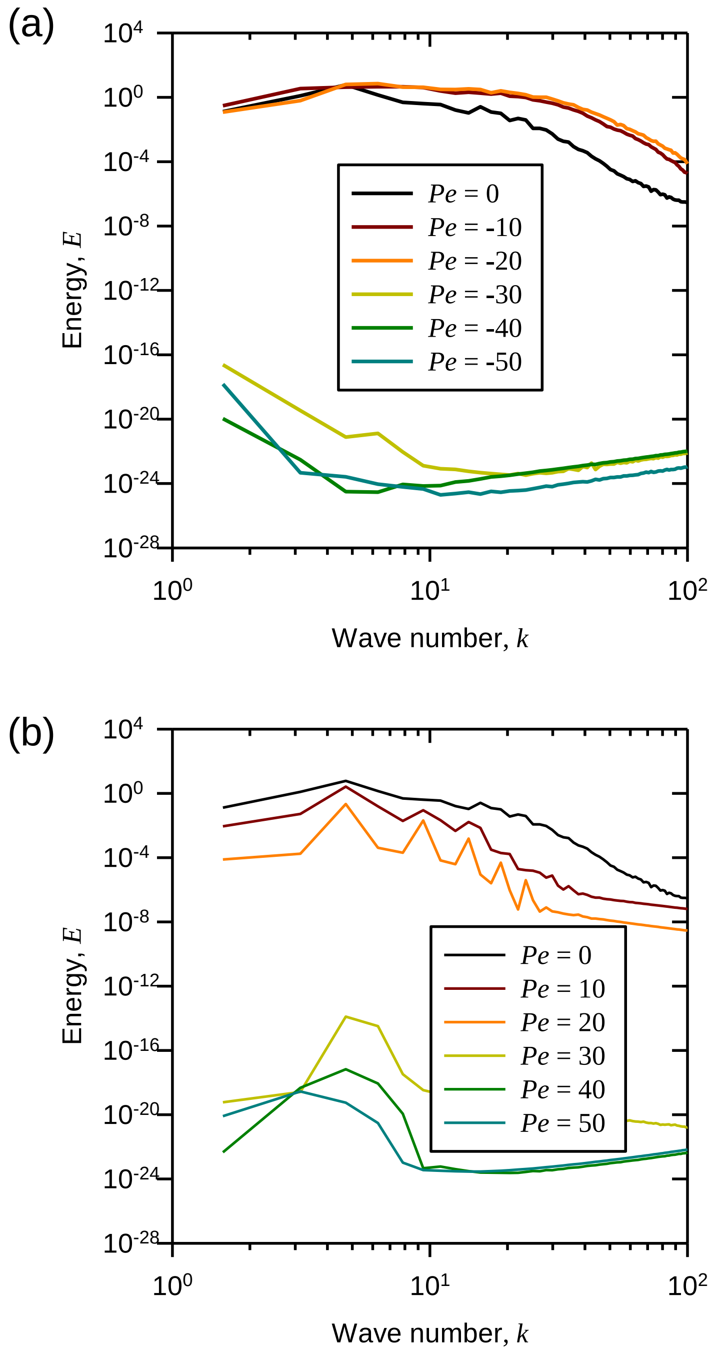

Figure S1. Energy spectrum of random vortices at the edge of the EDL is plotted according to the wave number when (**a**) the *Pe* was negative (downward flow) and (**b**) the *Pe* was positive (upward flow).
